# Sonographic Identification of Klippel-Trenaunay-Weber Syndrome

**DOI:** 10.1155/2013/595476

**Published:** 2013-12-03

**Authors:** Yigit Cakiroglu, Emek Doğer, Sule Yildirim Kopuk, Yasemin Dogan, Eray Calıskan, Gülseren Yucesoy

**Affiliations:** ^1^Department of Obstetrics and Gynecology, Kocaeli University School of Medicine, Kocaeli 41000, Turkey; ^2^Department of Obstetrics and Gynecology, Maternal-Fetal Unit, Kocaeli University School of Medicine, Kocaeli 41000, Turkey

## Abstract

Klippel-Trenaunay-Weber syndrome is a rare cutaneous vascular disorder characterized by the presence of multiple hemangiomata, arteriovenous fistulas, and limb hypertrophy. We report the prenatal sonographic findings in a case of Klippel-Trenaunay-Weber (KTW) syndrome including fetal limb hypertrophy and large subcutaneous cystic lesions. Prenatal diagnosis is possible by ultrasound examination and recognition important for prevention of complications and future management.

## 1. Introduction

Klippel-Trenaunay-Weber (KTW) syndrome is a rare cutaneous vascular disorder characterized by large cutaneous hemangiomas, malformations of the capillary, venous, and lymphatic vessels, and bony or soft tissue hypertrophy [1]. It is a rare, usually sporadic condition with death to live birth ratio of 1 : 100000 [2]. Prenatal diagnosis is important for treatment and prevention of complications. Herein is a report on a prenatal diagnosis of a 25-week gestation period, which subsequently led to the termination of a pregnancy.

## 2. Case Report

**Figure 1:**
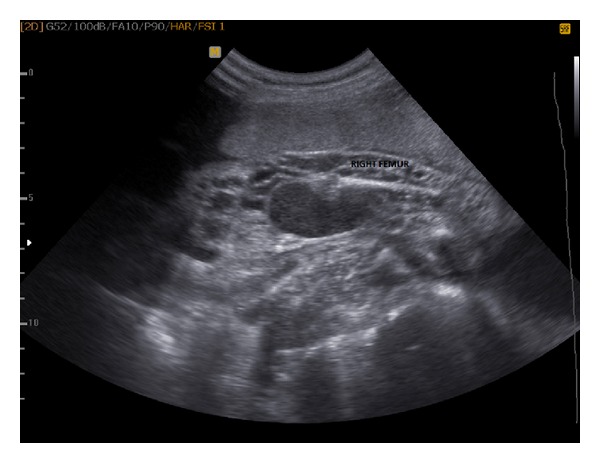
Longitudinal ultrasound image of the right thigh at 25 weeks and 4 days of gestation. Multiple cystic areas over the entire segment shown. This pattern continued below the knee, but the foot was not involved.

A 27-year-old gravida 1, para 0 pregnant woman at 25 weeks and 4 days of gestation was referred to our perinatology unit for a congenital anomaly scan because of suspected cystic structures around the right leg. The parents were nonconsanguineous and healthy. There was no family history of congenital malformations, nor was there any history of vascular disorders. Both two-dimensional (2D) and three-dimensional (3D) ultrasonographic examinations were performed using the Medison Sonoace 8x ultrasound machine, which revealed that the upper and lower parts of the right leg of the male fetus were grossly enlarged, due to numerous subcutaneous sonolucent multiloculated cystic lesions (Figure 1).

**Figure 2:**
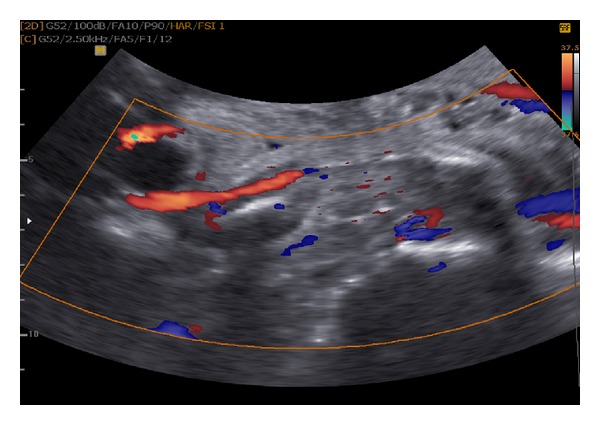
Color Doppler imaging (sagittal view) demonstrated prominent vessels surrounding multiple cystic structure.

Another test to examine the right leg more thoroughly used the Color Doppler examination, which revealed the presence of persistent embryonic lateral marginal veins (Figure 2). In addition, the test determined that there were no further structural defects and that there was a normal amniotic fluid compartment.

**Figure 3:**
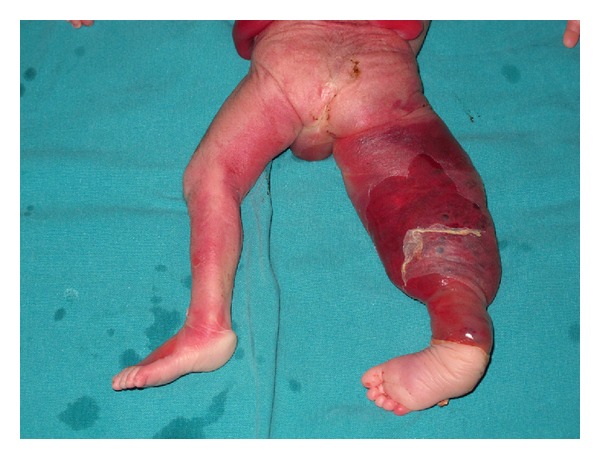
Macroscopic appearance of the hemangioma involving right thigh of the fetus.

The asymmetric limb hypertrophy and large subcutaneous cystic lesions suggested the presence of Klippel-Trenaunay-Weber syndrome and Proteus syndrome. Amniocentesis was performed and the cytogenetic result revealed a karyotype of 46, XY. The parents were counselled by a multiprofessional team regarding the possible outcomes and they elected to terminate the pregnancy. After a feticide procedure, the mother delivered a 1150-gram, ex-male fetus, which showed a hypertrophy of the lower right limb, the right half of the scrotum, and the lower trunk. A bluish discoloration along the limb with varicosities was also seen on the macroscopic view (Figure 3).

## 3. Discussion

Klippel-Trenaunay described KTW syndrome in 1900, which was characterized by a triad of a port wine stain, varicose veins and hemangiomata, and bony or soft tissue hypertrophy of an extremity [3]. First ultrasonographic prenatal diagnosis was defined in 1988 [4]. A majority of patients display all three features of the triad, and in most cases, nearly 100% of them have hemangiomata [1, 5]. The most common affected site is the leg [1]. The exact cause of the syndrome is unknown with both males and females affected equally [1, 6].

Recent theories suggest a mesodermal abnormality during fetal growth, abnormal regulation, or production of growth factors primarily affecting angiogenesis [7]. It is most likely, as speculated by Berry et al., that in KTS there is an alteration in vascular remodelling probably at the level of altered angiopoietin-2-antagonism [8, 9].

The venous malformations frequently present the persistence of embryonic veins of which the lateral marginal vein (the vein of Servelle) is the most typical finding. The vein originates from the lateral aspect of the foot and courses upwards along the lateral border of the leg [10].

Nonimmune hydrops fetalis, polyhydramnios, cardiac failure, macrocrania, ventriculomegaly, and hepatomegaly are other prenatal features [7, 11, 12]. High-output cardiac failure and coagulopathy could be caused by hemangioma-associated diffused intravascular coagulation and intravascular hemolysis [7].

In the presence of asymmetrical limb growth detected prenatally, diagnostic efforts are made to make differential diagnosis among KTW, Proteus syndrome, Beckwith-Wiedemann syndrome, neurofibromatosis, soft tissue sarcomas, lymphangioma, and so on.

The main differential diagnosis is the Proteus syndrome which is a rare condition and belongs to the complex of hamartomatoses. Major characteristic of the clinical picture is high variability. Hemihypertrophy, macrodactyly, exostoses, and subcutaneous masses of various types, such as lipomas, hamartomas, hemangiomas, lymphohemangiomas, and epidermal nevus, are major findings in more than one-half of the patients [13]. The course of the disease is characterized by an irregular progression with aggressive periods followed by quiet periods. In most cases, the major manifestations develop during infancy and are diagnosed after the first year.

The case presents a diagnosis of KTW syndrome showing an asymmetric limb hypertrophy of the right lower limb, subcutaneous cystic lesions, and the presence of lateral marginal vein.

In conclusion, KTW is a rare syndrome which is associated with life threatening complications like bleeding in the gastrointestinal tract, genitourinary system, spleen, liver, or central nervous system. Intermittent thrombophlebitis, thrombocytopenia, coagulation defects, severe anemia, congestive cardiac failure or autoamputations of toes are seen in patients as well [5]. Prenatal diagnosis is possible through ultrasound examinations, thus leading to the important recognition and prevention of complications, and for future management. Close monitoring to observe nonimmune hydrops fetalis, oligohydramnios, cardiomegaly, and polyhydramnios followed prompt delivery where intensive neonatal care facilities are available.
